# Lymphocyte Dynamics and the Emergence of Secondary Autoimmunity Following Immune Reconstitution Therapies in Multiple Sclerosis

**DOI:** 10.1212/NXI.0000000000200497

**Published:** 2025-10-16

**Authors:** Sofia Sandgren, Lenka Novakova, Markus Axelsson, Igal Rosenstein, Jan Nils Lycke, Clas Malmeström

**Affiliations:** 1 Department of Clinical Neuroscience, Institute of Neuroscience and Physiology at Sahlgrenska Academy, University of Gothenburg, Gothenburg, Sweden;; 2 Sahlgrenska, Department of Neurology, Region Västra Götaland, Gothenburg, Sweden; and; 3 Laboratory for Clinical Immunology, Sahlgrenska University Hospital, Gothenburg, Sweden.

## Abstract

**Background and Objectives:**

Understanding the immunologic changes induced by immune reconstitution therapies (IRTs) is key to optimizing multiple sclerosis (MS) treatment. We evaluate lymphocyte dynamics and their association with secondary autoimmune disease (SAD) and its recurrence after treatment with alemtuzumab (ALZ), autologous hematopoietic stem cell transplantation (AHSCT), and cladribine tablets (CladT).

**Methods:**

People with MS (pwMS) initiating treatment with ALZ, AHSCT, and CladT were included in this cohort study. Blood samples were collected at baseline (BL) and at months (M) 6, 12, and 24 for flow cytometry analysis of lymphocyte subpopulation.

**Results:**

A total of 130 pwMS (ALZ: n = 51; AHSCT: n = 20; CladT: n = 59) were included, with a mean (SD) age of 35.5 (±8.2) years. The median (IQR) Expanded Disability Status Scale (EDSS) score was 1.5 (1.0–2.5) at BL. The median follow-up duration was 4.7 (ALZ: 5.0; AHSCT: 4.2; CladT: 3.7) years. During follow-up, 29.2% (38/130; ALZ n = 29; AHSCT n = 3; CladT n = 6) received a SAD diagnosis and 43.1% (56/130; ALZ n = 15; AHSCT n = 13; CladT n = 28) showed no disease activity. The SAD cohort (n = 38) showed lower median BL ratios of CD4^+^ T-cell recent thymic emigrant (Trte):CD4 (0.26 [0.12–0.34], *p* = 0.04) and CD4^+^ Trte:CD8+ terminally differentiated effector memory (Temra) (1.73 [0.76–4.39], *p* = 0.02) compared with the non-SAD cohort (CD4^+^ Trte:CD4 = 0.31 [0.23–0.42]; CD4^+^ Trte:CD8+ Temra = 3.61 [1.47–7.24]). During follow-up, the SAD cohort exhibited a greater relative increase in CD4^+^ Trte:CD4, CD4^+^ Trte:CD8+ Temra, and CD4^+^ T regulatory cell (Treg):CD8+ Temra ratios at M12 and M24, compared with BL, relative to the non-SAD group. The difference at M24 was most pronounced for the CD4^+^ Trte:CD8+ Temra ratio (SAD: +100% vs non-SAD: −23%, *p* < 0.001), with this difference being confirmed in the ALZ cohort (SAD: +123% vs non-SAD: −21%, *p* = 0.03), but not in the CladT cohort (SAD: −62% vs non-SAD: −38%, *p* = 0.39). In a multivariable Cox analysis, BL CD8^+^ T-cell count (aHR 0.34, 95% CI 0.15–0.80, *p* = 0.01) was associated with a reduced hazard of evidence of disease activity.

**Discussion:**

A low BL CD4^+^ Trte:CD8^+^ Temra ratio, accompanied by a sharp relative increase during follow-up, was associated with SAD development. While no definitive associations were found between BL lymphocyte subpopulations and disease activity, a lower CD8^+^ T-cell count may suggest an increased risk.

## Introduction

The traditional treatment of multiple sclerosis (MS), which involves chronic immunosuppression and subsequently increased susceptibility to infections and risk of malignancy, is challenged by modern immune reconstitution therapies (IRTs). Three therapies are considered IRTs for relapsing-remitting MS (RRMS): alemtuzumab (ALZ), autologous hematopoietic stem cell transplantation (AHSCT), and cladribine tablets (CladT).

IRTs are administered once (AHSCT) or intermittently as short courses (ALZ and CladT), leading to transient immunosuppression, followed by a “reboot” of the immune system with the goal of eradicating previous autoimmunity. This approach enables long-term disease control, even during treatment-free follow-up, while reducing safety risks over time.

However, IRTs carry risks, with secondary autoimmune disease (SAD) being one of the most important. Although ALZ and AHSCT are both associated with an increased risk of SAD, this has not been observed during CladT treatment.^1,2^ Long-term follow-up of ALZ treatment shows that 48% of patients develop SAD. The most common SAD is autoimmune thyroid disease (AITD), occurring in 30% of ALZ-treated and 8.3% of AHSCT-treated patients.^3,4^

Understanding the immunologic changes induced by IRTs and their association with clinical outcomes, including the development of SAD, is crucial for optimizing their future use in MS treatment. The aim of this study was to compare lymphocyte subset dynamics after treatment with ALZ, AHSCT, and CladT and their correlation with disease activity recurrence and SAD.

## Methods

### Study Population

This observational cohort study prospectively enrolled patients with RRMS who started treatment with ALZ, AHSCT, and CladT during years 2014–2016, 2014–2022, and 2018–2021, respectively, at the MS Centre, Sahlgrenska University Hospital, Gothenburg, Sweden. Eligible patients were aged 18 years or older and could be either treatment-naive or previously treated. Observational data were reassessed retrospectively; included patients fulfilled the 2017 revised McDonald criteria for RRMS.^5^ Baseline (BL) was the date of treatment start. Follow-up was the date of data extraction: 18.09.2021 (ALZ), 18.05.2024 (AHSCT), and 25.05.2024 (CladT). For this article, the STROBE reporting guidelines for observational studies were followed.^6^

### Data Collection and Assessments

Demographic data (age, sex), MS history (onset date, diagnosis date, prior use of disease-modifying therapies, number of relapses in the year prior to BL), and BL characteristics (Expanded Disability Status Scale [EDSS] score,^7^ T2 lesion load, and presence of gadolinium-enhancing lesions on MRI) were collected for each patient. Electronic health records were further reviewed for evidence of any preexisting autoimmune disorder other than MS. The date and number of ALZ infusions and CladT courses were documented, along with yearly clinical (EDSS score, occurrence of relapses [defined according to the revised 2017 McDonald criteria],^5^ and SAD [details on the surveillance and definition of SADs are provided in the eMethods] based on chart review) and imaging (MRI) data during follow-up. Blood samples were collected at BL and every 3 months up to and including month 24 and were analyzed for absolute lymphocyte counts and their subsets.

No evidence of disease activity (NEDA-3) was defined as no new or enlarging MRI lesions, no relapses, and no confirmed disability worsening (CDW) event,^8^ whereas relapse, MRI activity, or disability worsening were considered as evidence of disease activity (EDA-3). CDW was defined as a 1-point increase in EDSS for BL scores of 1–5, a 0.5 increase in BL scores of 5.5 or higher, and an increase of 1.5 in the BL score of 0, confirmed after at least 6 months.^9^

### Flow Cytometry T-Cell Panel of Peripheral Blood and T-Cell Receptor Excision Circles

Flow cytometry was performed using 2 tubes. Tube 1 (TBNK TrueCount, BD) was used to determine absolute lymphocyte counts, including CD3, CD16/56, CD45, CD4, CD19, and CD8, according to the manufacturer's instruction and the standard operational procedures of the Immunology laboratory, Sahlgrenska University Hospital. Tube 2 was used for more detailed T-cell phenotypning, including CD3, CD4, CD8, CD45RA, CD45, CD25, CD127, and CD197. Analyses were performed on fresh specimens using a FACS Lyric flow cytometry instrument (BD). Fluorochrome selection and gating strategies followed the established protocol.^10^

T-cell receptor excision circles (TRECs) were measured in molecules per 10 × 10^6^ cells according to an in-house RT-PCR method (Light Cycler, Roche) consisting of 2 TREC primers and 2 GAPDH primers and in-house–produced plasmid from Plasmid pCR2.1-human TREC, gene length 157bp (Eurofins MWG/Operon), and calibrator from a pool of umbilical cord blood.

### Statistical Analysis

Demographic and clinical characteristics were described as counts and percentages, mean and SD, and median and interquartile range [IQR], as appropriate. Visual inspection of histograms and the Shapiro-Wilk test were performed to determine whether BL variables had a normal distribution (only age was normally distributed). Between-group comparisons involving more than 2 groups were conducted using the Fisher exact test or the χ^2^ test for categorical variables and either 1-way between-group ANOVA or the Kruskal-Wallis test for continuous (or ordinal) variables, as appropriate based on data distribution. For the Kruskal-Wallis test, post hoc pairwise comparisons were performed with Bonferroni-adjusted *p* values to control for multiple testing.

Demographic analyses included all patients (n = 130). For analyses of lymphocyte dynamics, T-cell subpopulations, SAD vs non-SAD comparisons, and Cox models for EDA-3 prediction, patients with BL lymphocyte counts below 0.8 × 10e9/L (i.e., World Health Organization [WHO] grade 2 lymphopenia; n = 13) were excluded. In addition, for analyses involving the variables total lymphocytes, CD19^+^ B cells, and CD3:CD19 ratio, patients who met the lymphocyte cutoff but had received B-cell depleting therapy as the last disease-modifying therapy (DMT) before IRT (10 CladT) were excluded. An additional 13 patients who met the lymphocyte cutoff and had no recent B-cell depleting therapy had missing BL total lymphocyte data (eTable 1 provides details). Missing lymphocyte values at months 6, 12, and 24 were replaced with values from months 3 (n = 5), 9 (n = 4), and 18 or 21 (n = 10), respectively, when available. We calculated the following T-cell subpopulation ratios at BL and during follow-up at months 6, 12, and 24: CD4^+^ T regulatory cell (Treg):CD4, CD4^+^ T-cell recent thymic emigrant (Trte):CD4, CD8^+^ terminally differentiated effector memory (Temra):CD8, CD4^+^ Trte:CD8^+^ Temra, and CD4^+^ Treg:CD8^+^ Temra. These ratios were used to examine changes in the proportion of different T-cell subpopulations relative to the total CD4^+^, CD8^+^, and CD8^+^ Temra T-cell populations, respectively, and to investigate whether these changes were associated with the development of SAD. The Mann-Whitney *U* test was used to compare continuous variables between 2 groups (SAD vs non-SAD), based on non-normal distribution of data. Cox regression analysis was used to define the association of lymphocyte subsets and T-cell subpopulation ratios with the time to evidence of disease activity (i.e., EDA-3). Results were reported as adjusted hazard ratio (aHR) with a corresponding 95% confidence interval (CI). For the EDA-3 end point, we adjusted for the following potential confounding factors: age, sex, disease duration, MRI T2 lesion burden, and number of previous DMTs. Given the exploratory nature of these Cox models, no correction for multiple comparisons was applied. SPSS version 28.00 (IBM, NY) and GraphPad Prism 10.0.3 (GraphPad Inc., CA) were used for statistical analyses. All tests were 2-sided, with significance threshold *p* < 0.05.

### Standard Protocol Approvals, Registrations, and Patient Consents

The study was approved by the Regional Ethics Review board in Gothenburg (reference number 460-13, 02.10.2013; 895-13, 13.01.2014; and 2020-06766, 21.01.2019). All patients gave written informed consent.

### Data Availability

The corresponding author will review written requests for access to the data presented in this article and will determine the appropriateness of its use. If the use is appropriate, a data sharing agreement will be put in place before a fully deidentified version of the data set used for the analysis with individual participant data is made available.

## Results

### Study Population

The cohort consisted of 130 people with MS (pwMS; 39.2% treated with ALZ, 15.4% treated with AHSCT, and 45.4% treated with CladT), with a mean (SD) age of 35.5 (±8.2) years and a median (IQR) EDSS score of 1.5 (1.0, 2.5) at BL. Details of the BL demographic and clinical characteristics are provided in Table 1. The Kruskal-Wallis test indicated significant differences in T2 lesion load across the ALZ, AHSCT, and CladT cohorts (*p* < 0.001). Post hoc pairwise comparisons with Bonferroni correction revealed that the ALZ and AHSCT cohorts had significantly higher T2 lesion load than the CladT cohort (*p* < 0.001, respectively; *p* = 0.003). However, no difference was found in contrast-enhancing lesions across cohorts. Furthermore, cohorts differed in disability (*p* = 0.002) and prior DMT exposure (*p* < 0.001) at BL. Post hoc Bonferroni-corrected comparisons showed that the AHSCT cohort had the highest disability (vs ALZ: *p* = 0.045; CladT: *p* = 0.001) while the CladT cohort included the fewest patients with prior DMTs (vs ALZ: *p* = 0.005; AHSCT: *p* = 0.003). When BL DMTs were categorized by mechanism of action, differences emerged between the IRT groups (*p* = 0.014): very late antigen 4 (VLA-4) therapies were the most commonly used overall, although least frequent in the CladT group, which had the highest proportion of prior anti-CD20 treatment.

**Table 1:** Study Population Characteristics and Demographics

	Total cohort (n = 130)	ALZ (n = 51)	CladT (n = 59)	AHSCT (n = 20)	*p* Value
Sex = Women	92 (71)	31 (61)	48 (81)	13 (65)	0.05^a^
Age (y), mean ± SD	35.5 ± 8.2	36.0 ± 7.1	35.4 ± 9.0	34.2 ± 8.4	0.68^b^
Disease duration (y)	5.3 (2.5–9.4)	5.6 (2.6–11.2)	5.5 (1.2–8.7)	3.9 (2.8–7.7)	0.47^c^
Diagnosis duration (y)	2.9 (0.6–7.4)	4.1 (1.1–10.0)	1.3 (0.2–6.6)	2.8 (1.5–6.1)	0.04^c^
No. of relapses <1 y before BL	1.0 (0.0–1.0)	0.0 (0.0–1.0)	1.0 (0.0–1.0)	1.0 (0.3–1.8)	0.01^c^
BL EDSS score	2.0 (1.0–2.5)	2.0 (1.0–2.5)	1.5 (0.0–2.0)	2.5 (2.0–3.5)	0.002^c^
No. of previous DMTs	2.0 (1.0–3.0)	2.0 (1.0–3.0)	1.0 (0.0–2.0)	2.0 (1.0–3.0)	<0.001^c^
Treatment naive	26 (20)	7 (14)	19 (32)	0 (0)	0.002^a^
DMT at BL					<0.001^a^
Anti-CD20	12 (9)	1 (2)	10 (17)	1 (5)	
Anti-CD52	3 (2.5)	0 (0)	0 (0)	3 (15)	
Anti–VLA-4	61 (47)	33 (64)	17 (30)	11 (55)	
S1P modulators	8 (6)	4 (8)	2 (3)	2 (10)	
Enhances NrF2, reducing free radical activity	9 (7)	2 (4)	7 (12)	0 (0)	
Shift to anti-inflammatory Th2 response	4 (3)	2 (4)	2 (3)	0 (0)	
Prevent proliferation of autoimmune T and B cells	4 (3)	2 (4)	2 (3)	0 (0)	
Synthetic chlorinated deoxyadenosine analog	3 (2.5)	0 (0)	0 (0)	3 (15)	
No. of T2 lesions at BL					<0.001^c^
1–9	33 (25)	4 (8)	26 (44)	3 (15)	
10–20	31 (24)	12 (23)	16 (27)	3 (15)	
>20	66 (51)	35 (69)	17 (29)	14 (70)	
No. of patients with ≥1 T1 Gd + lesion at BL	41 (32)	15 (29)	19 (32)	7 (35)	0.93^c^
Follow-up (y)	4.7 (3.4–5.0)	5.0 (4.9–5.1)	3.7 (3.1–4.8)	4.2 (2.6–6.2)	<0.001^c^

Abbreviations: AHSCT = autologous hematopoietic stem cell transplantation; ALZ = alemtuzumab; BL = baseline; CladT = cladribine tablets; DMTs = disease-modifying therapies; EDSS = Expanded Disability Status Scale; Gd = gadolinium; No. = number; NrF2 = nuclear factor erythroid 2 related factor 2; VLA-4 = very late antigen 4.

Reported values are n (%) and median (IQR) if not stated otherwise. For group comparisons, the following were applied as appropriate:

a Fisher exact test or χ^2^ test

b One-way between-group ANOVA

c Kruskal-Wallis test.

Anti-CD20: rituximab; anti-CD52: ALZ; anti–VLA-4: natalizumab; S1P modulators: fingolimod; enhances NrF2, reducing free radical activity: dimethyl fumarate; shift to anti-inflammatory Th2 response: beta interferon and glatiramer acetate; prevent proliferation of autoimmune T and B cells: teriflunomide; synthetic chlorinated deoxyadenosine analog: CladT.

The median follow-up was 4.7 (ALZ: 5.0; AHSCT: 4.2; CladT: 3.7) years. During follow-up, 21.5% (28/130; ALZ, n = 13; AHSCT, n = 3; CladT, n = 12) switched to another DMT, 43.1% (56/130; ALZ, n = 15; AHSCT, n = 13; CladT, n = 28) met NEDA-3, and 29.2% (38/130; ALZ, n = 29; AHSCT, n = 3; CladT, n = 6) received a SAD diagnosis. The median (IQR) time interval between IRT initiation and SAD occurrence was 23.5 (15–29) months (ALZ: 23 [13–27]; CladT: 35 [23–43]; AHSCT: 22 [20–25]). eTable 2 presents the time from IRT initiation to SAD diagnosis and the corresponding SAD type for each case.

#### Lymphocyte Dynamics

**Figure 1:**
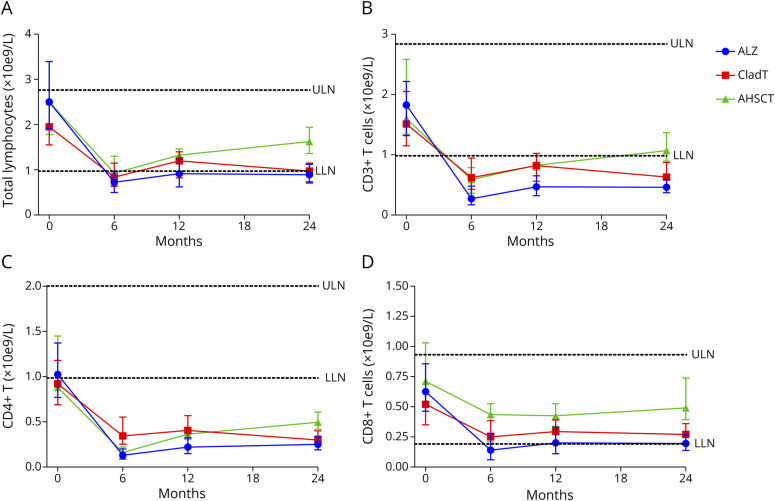
Median Values of Total Lymphocytes and T-Cell Subpopulations in pwMS Treated With ALZ, CladT, and AHSCT The figure shows median (IQR) values of (A) total lymphocytes, (B) CD3^+^ T cells, (C) CD4^+^ T cells, and (D) CD8^+^ T cells in the ALZ cohort (blue dots), CladT cohort (red squares), and AHSCT cohort (green triangles). Month 0 = BL. Horizontal dotted lines represent ULN and LLN. ALZ = alemtuzumab; AHSCT = autologous hematopoietic stem cell transplantation; BL = baseline; CladT = cladribine tablets; IQR = interquartile range; LLN = lower limit of normal; pwMS = people with multiple sclerosis; ULN = upper limit of normal.

**Figure 2:**
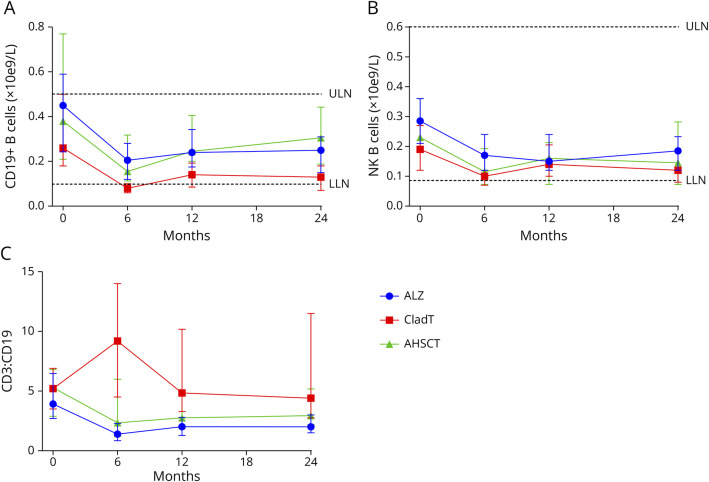
Median Values of B and NK Cells and CD3:CD19 Ratio in pwMS Treated With ALZ, CladT, and AHSCT The figure shows median (IQR) values of (A) CD19^+^ B cells, (B) NK cells, and (C) CD3:CD19 in the ALZ cohort (blue dots), CladT cohort (red squares), and AHSCT cohort (green triangles). Month 0 = BL. Horizontal dotted lines represent ULN and LLN. ALZ = alemtuzumab; AHSCT = autologous hematopoietic stem cell transplantation; BL = baseline; CladT = cladribine tablets; IQR = interquartile range; LLN = lower limit of normal; NK = natural killer; pwMS = people with multiple sclerosis; ULN = upper limit of normal.

Total lymphocytes, CD3^+^ and CD8^+^ T cells, CD19^+^ B cells, and natural killer (NK) cells were within the normal range at BL for all 3 IRT cohorts while the median (IQR) CD4^+^ T-cell values for CladT (0.92 [0.69–1.18]) and AHSCT (0.88 [0.69–1.45]) cohorts were slightly below the normal range (1.0–2.1 × 10e9/L). However, no statistically significant difference in the median CD4^+^ T-cell value was observed at BL across IRT groups (Figure 1, A–D and Figure 2, A–C). All IRT cohorts exhibited a decrease in total lymphocytes and subpopulations during follow-up, with notable differences observed between IRT cohorts. The relative changes from BL to months 6, 12, and 24, presented separately for each IRT group, are given in Table 2 and eTable 3.

**Table 2:** Median Percentage Change From BL in Lymphocytes and T-Cell Subpopulations

	ALZ				CladT				AHSCT			
	BL	M6	M12	M24	BL	M6	M12	M24	BL	M6	M12	M24
	Median (IQR)				Median (IQR)				Median (IQR)			
Lymphocytes												
Lymphocytes tot. (×10e9/L)	2.50 (1.94–3.40), n = 44	−75%	−63%	−63%	1.95 (1.47–2.56), n = 35	−53%	−44%	−58%	2.50 (1.78–3.39), n = 15	−61%	−48%	−38%
CD3^+^ T cells (×10e9/L)	1.83 (1.37–2.22), n = 44	−86%	−76%	−72%	1.51 (1.09–2.10), n = 43	−56%	−48%	−60%	1.60 (1.34–2.58), n = 15	−62%	−51%	−38%
CD4^+^ T cells (×10e9/L)	1.03 (0.80–1.32), n = 44	−89%	−79%	−75%	0.92 (0.69–1.18), n = 43	−60%	−53%	−67%	0.88 (0.69–1.45), n = 15	−84%	−67%	−58%
CD8^+^ T cells (×10e9/L)	0.66 (0.48–0.86), n = 44	−81%	−73%	−70%	0.51 (0.34–0.62), n = 43	−46%	−43%	−54%	0.71 (0.50–1.03), n = 15	−45%	−34%	−8%
NK cells (×10e9/L)	0.30 (0.23–0.37), n = 44	−34%	−48%	−41%	0.19 (0.12–0.27), n = 43	−39%	−28%	−36%	0.23 (0.18–0.36), n = 15	−47%	−33%	−47%
CD19^+^ B cells (×10e9/L)	0.47 (0.25–0.60), n = 44	−60%	−47%	−48%	0.26 (0.18–0.50), n = 35	−78%	−50%	−58%	0.38 (0.21–0.77), n = 15	−61%	−27%	−16%
CD3:CD19	3.90 (2.70–6.08), n = 44	−70%	−53%	−57%	5.20 (3.50–6.90), n = 35	63%	1.5%	0.0%	5.30 (2.90–6.80), n = 15	−41%	−40%	−44%
T-cell subpopulation ratios												
CD4^+^ Trte:CD4	0.27 (0.16–034), n = 42	−27%	14%	20%	0.36 (0.23–0.46), n = 43	−11%	−5%	−13%	0.25 (0.14–0.31), n = 15	−21%	63%	47%
CD4^+^ Trte:CD8+ Temra	2.04 (0.89–1.15), n = 41	−63%	−5%	60%	5.04 (1.85–9.90), n = 43	−40%	−42%	−44%	1.55 (0.52–3.37), n = 15	−75%	49%	71%
CD4^+^ Treg:CD8+ Temra	0.78 (0.31–1.49), n = 41	−23%	−11%	5%	1.23 (0.61–2.35), n = 43	4%	−21%	−16%	0.48 (0.21–1.29), n = 15	−63%	−27%	−19%

Abbreviations: AHSCT = autologous hematopoietic stem cell transplantation; ALZ = alemtuzumab; BL = baseline; CladT = cladribine tablets; IQR = interquartile range; M = month; NK = natural killer; Temra = terminally differentiated effector memory; tot. = total; Treg = T regulatory cell; Trte = T-cell recent thymic emigrant.

A full version of this table is given in eTable 3.

All IRT groups showed a significant decrease in their total lymphocyte counts at 6 and 12 months, compared with BL (Figure 1A). Subnormal levels persisted in ALZ and CladT cohorts during follow-up. At month 24, post hoc Bonferroni-corrected comparisons showed that both ALZ (median [IQR]: 0.92 [0.72–1.20] × 10e9/L; *p* < 0.001) and CladT (0.96 [0.73–1.16], *p* < 0.001) cohorts were associated with lower total lymphocyte counts, compared with the AHSCT cohort (1.63 [1.36–1.94]). The AHSCT cohort had normalized total lymphocyte counts at month 24.

CD3^+^, CD4^+^, and CD8^+^ T cells were significantly decreased in all IRT groups (Figure 1, B–D). The reduction was most pronounced in the ALZ group, which showed significantly lower median (IQR) CD3^+^ (month 6: ALZ: 0.26 [0.17–0.43]; AHSCT: 0.59 [0.36–0.79], *p* = 0.001; CladT: 0.62 [0.45–0.92], *p* < 0.001; month 12: ALZ: 0.40 [0.31–0.63]; AHSCT: 0.83 [0.75–0.99], *p* = 0.001; CladT: 0.80 [0.56–1.02], *p* < 0.001), CD4^+^ (month 12: ALZ: 0.22 [0.15–0.29]; AHSCT: 0.37 [0.31–0.42]; *p* = 0.009; CladT: 0.41 [0.31–0.57], *p* < 0.001), and CD8^+^ (month 6: ALZ: 0.13 [0.05–0.27]; AHSCT: 0.44 [0.21–0.53], *p* < 0.001; CladT: 0.25 [0.16–0.37], *p* = 0.001) T-cell counts compared with the AHSCT and CladT groups, based on post hoc Bonferroni-corrected comparisons. CladT-treated patients had least affected CD4^+^ T-cell counts at month 6 (CladT: median [IQR]: 0.35 [0.26–0.55]; vs ALZ: 0.12 [0.09–0.18], *p* < 0.001; AHSCT: 0.16 [0.12–0.22], *p* < 0.001), but the recovery was slow. By contrast, patients treated with AHSCT recovered their CD3^+^ (AHSCT: median [IQR]: 1.07 [0.89–1.37]; vs ALZ: 0.46 [0.36–0.59], *p* < 0.001; CladT: 0.60 [0.45–0.80], *p* = 0.006), CD4^+^ (AHSCT: 0.50 [0.42–0.61]; vs ALZ: 0.25 [0.19–0.34], *p* < 0.001; CladT: 0.30 [0.23–0.40], *p* = 0.014), and CD8^+^ (AHSCT: 0.49 [0.39–0.74]; vs ALZ: 0.20 [0.12–0.29], *p* < 0.001; CladT: 0.26 [0.16–0.35], *p* = 0.002) T-cell counts faster and had higher cell counts at month 24 compared with the ALZ and CladT cohorts.

No difference in the median CD19^+^ B-cell count was observed at BL across the 3 IRT cohorts. The CD19^+^ B-cell count was significantly reduced in all IRT groups at month 6 (Figure 2A). The reduction was most pronounced, and repopulation was slowest, in the CladT cohort. At months 6 (CladT: median [IQR]: 0.08 [0.05–0.10]; ALZ: 0.21 [0.14–0.28], *p* < 0.001; AHSCT: 0.16 [0.11–0.32], *p* = 0.001), 12 (CladT: 0.14 [0.07–0.22]; ALZ: 0.24 [0.18–0.32], *p* < 0.002; AHSCT: 0.25 [0.20–0.41], *p* = 0.004), and 24 (CladT: 0.13 [0.07–0.19]; ALZ: 0.25 [0.16–0.32], *p* < 0.001; AHSCT: 0.31 [0.19–0.44], *p* = 0.001), CD19^+^ B-cell levels in the CladT cohort were significantly lower than those in both the ALZ and AHSCT cohorts, as determined by post hoc Bonferroni-corrected comparisons.

NK cell counts were significantly reduced in all IRT groups (Figure 2B); however, the median value remained within the normal range (0.09–0.6 ×10e9/L) throughout the follow-up in all treatment groups.

The median CD3:CD19 ratio was similar across ALZ, CladT, and AHSCT cohorts at BL (Figure 2C). At month 6, the CladT cohort (median [IQR]: 9.50 [4.28, 13.5]) had a higher CD3:CD19 ratio than ALZ (1.3 [0.75–2.00], *p* < 0.001) and AHSCT (2.4 [1.20–5.60], *p* = 0.004) cohorts. The ratio remained higher than in the ALZ cohort at months 12 (CladT: 4.60 [3.20–9.90]; ALZ: 1.93 [1.23–2.66], *p* < 0.001) and 24 (CladT: 3.80 [2.60–9.85]; ALZ: 2.01 [1.48–2.79], *p* < 0.001).

### T-Cell Subpopulations

The relative reduction, given in eTable 3, of CD4^+^ T-cell subsets, Trte, Treg, Temra, T-cell naive (Tnaive), T-cell effector memory (Tem), and T-cell central memory (Tcm), at month 6, compared with BL, was most pronounced in the ALZ cohort, followed by the AHSCT cohort, and least affected in the CladT cohort. At month 24, some recovery was observed in the ALZ cohort, although this cohort still exhibited the greatest reduction. Recovery at 24 months was more pronounced in the AHSCT cohort. By contrast, the CladT cohort showed either no change or a slight further reduction.

Similarly, the 6-month reductions in CD8^+^ T-cell subsets (Temra, Tnaive, Tem, Tcm) were most pronounced in the ALZ cohort, followed by the AHSCT cohort, with the least reduction observed in the CladT cohort (eTable 3). Again, at month 24, the ALZ cohort showed signs of partial recovery in several subsets (e.g., Tnaive recovered from −93% to −68% and Tcm from −95% to −87%), although it still displayed the most substantial overall reduction. At 24 months, some recovery was also observed in the AHSCT cohort. By contrast, the CladT cohort showed no evidence of recovery between 6 and 24 months, with either stable or further declining levels (e.g., Temra declined from −21% to −45% and Tnaive remained unchanged at 75%). Thus, although the magnitude of initial reduction was smaller in the CladT cohort, the longitudinal data indicate a lack of recovery over time.

### Cross-Cohort Analyses

#### Role of Lymphocyte Subpopulations in the Association With SAD

**Figure 3:**
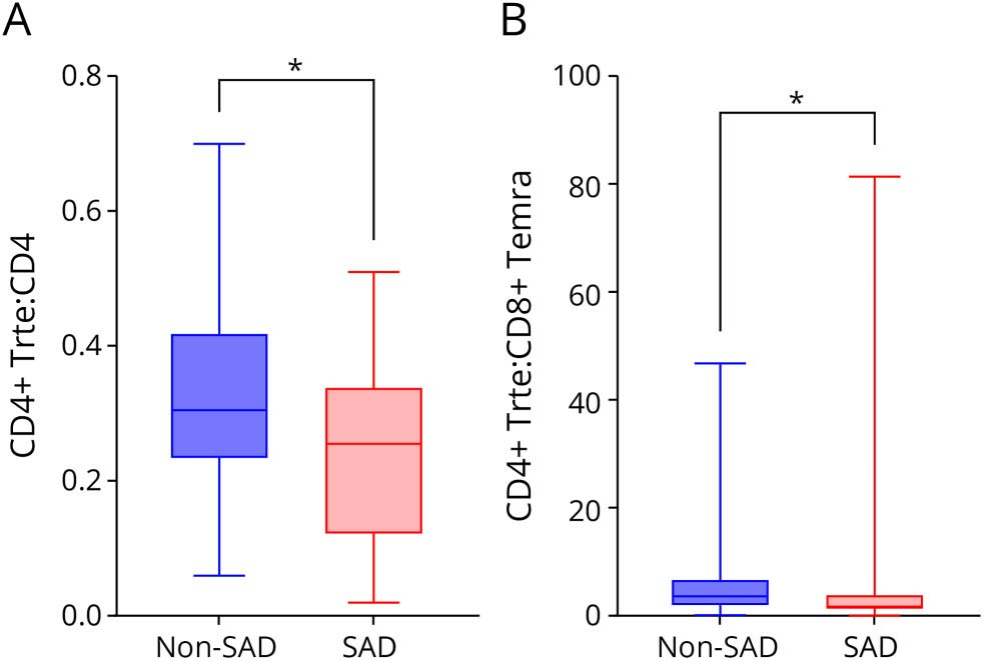
T-Cell Subpopulation Ratios at BL, SAD vs No-SAD Cohorts Each box plot shows the median (horizontal line), IQR (box), and range within whiskers. Panel A shows the CD4^+^ Trte:CD4^+^ ratio, and panel B shows the CD4^+^ Trte:CD8^+^ Temra ratio at BL, calculated from raw BL values and grouped by SAD vs no-SAD status. Statistical comparison was performed using the Mann-Whitney *U* test. *p < 0.05. BL = baseline; IQR = interquartile range; M = month; SAD = secondary autoimmune disease; Temra = terminally differentiated effector memory; Trte = T-cell recent thymic emigrant.

**Figure 4:**
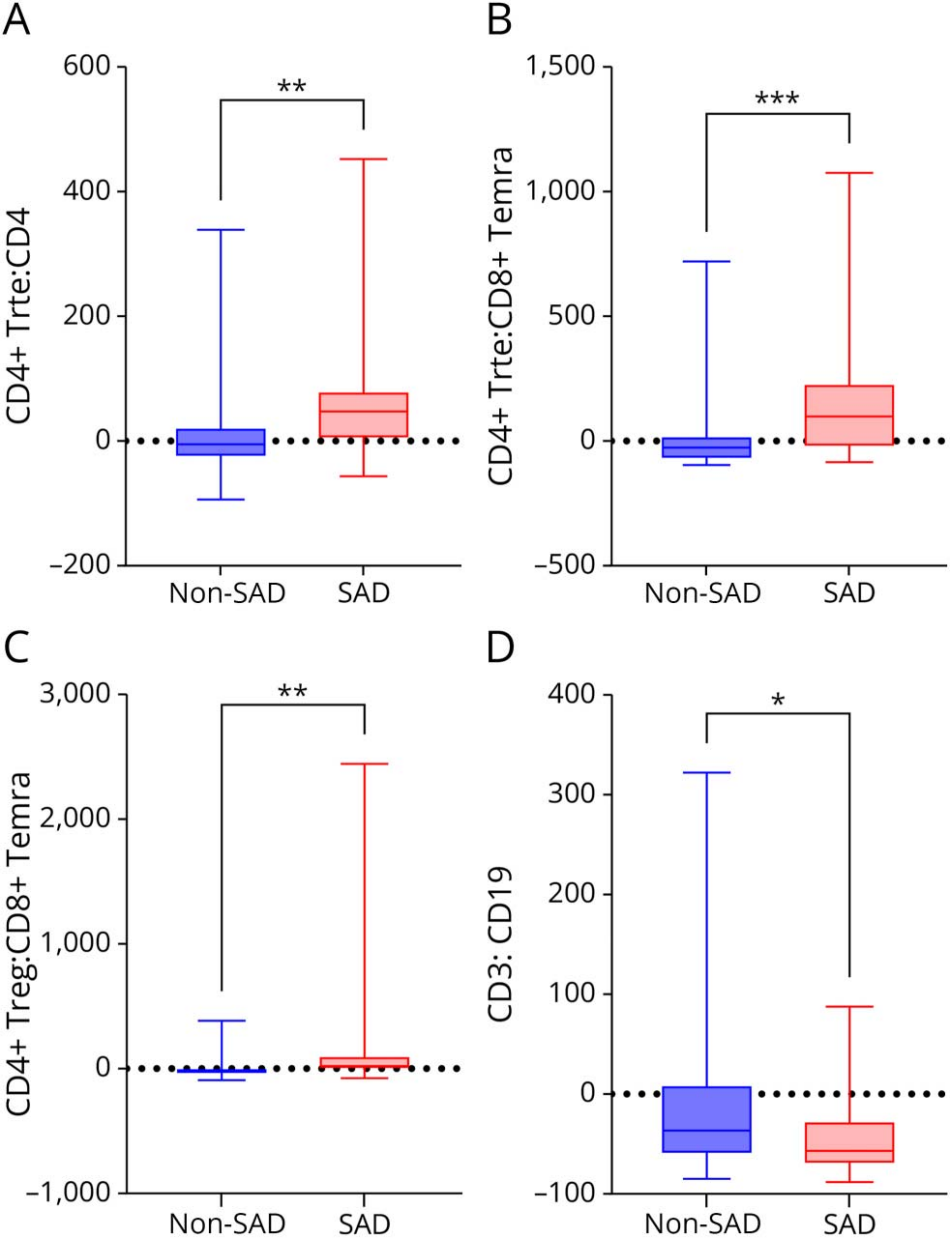
Percentage Change in T-Cell Subpopulation Ratios at Month 24, SAD vs Non-SAD Cohorts Each box plot shows the median (horizontal line), IQR (box), and range within whiskers. Panel A shows the CD4^+^ Trte:CD4 ratio, panel B shows the CD4^+^ Trte:CD8^+^ Temra ratio, panel C shows the CD4^+^ Treg: CD8^+^ Temra ratio, and panel D shows the CD3:CD19 ratio at M24, calculated from relative changes compared with BL and grouped by SAD vs no-SAD status. Statistical comparison was performed using the Mann-Whitney *U* test. *p < 0.05, **p < 0.01, ***p < 0.001. BL = baseline; IQR = interquartile range; M = month; SAD = secondary autoimmune disease; Temra = terminally differentiated effector memory; Treg = T regulatory cell; Trte = T-cell recent thymic emigrant.

At BL, pwMS who developed a SAD during follow-up exhibited lower median (IQR) CD4^+^ Trte:CD4 (0.26 [0.12–0.34], *p* = 0.04) and CD4^+^ Trte:CD8+ Temra (1.73 [0.76–4.39], *p* = 0.02) ratios compared with those who did not develop a SAD (CD4^+^ Trte:CD4 = 0.31 [0.23–0.42]; CD4^+^ Trte:CD8+ Temra = 3.61 [1.47–7.24]) (Table 3, eTable 4, Figure 3, A and B). By contrast, during follow-up, the SAD group exhibited a more reactive pattern, with a significantly greater relative increase in CD4^+^ Trte:CD4, CD4^+^ Trte:CD8+ Temra, and CD4^+^ Treg:CD8+ Temra ratios at months 12 and 24, compared with BL, relative to the non-SAD group (Table 3, eTable 4, Figure 4, A–C). The difference at month 24 was most pronounced for the CD4^+^ Trte:CD8+ Temra ratio (SAD: median [IQR]: 100% [−29 to 236] vs non-SAD: −23% [−77 to 25], *p* < 0.001), with this difference being confirmed in the ALZ cohort (SAD: 123% [41–327] vs non-SAD: −21% [−68 to 50], *p* = 0.03), but not in the CladT cohort (SAD: −62% [−84 to −24] vs non-SAD: −38%, [−77 to −2], *p* = 0.39). Furthermore, the CD3:CD19 ratio decreased significantly in the SAD cohort compared with the non-SAD cohort at 6 (−68% [−82 to −46] vs −10% [−67 to 68], *p* < 0.01), 12 (−51% [−74 to −39] vs −33% [−52 to 36], *p* = 0.03), and 24 (−57% [−71 to −19] vs −34% [−58 to 16], *p* = 0.03) months (Figure 4 D).

**Table 3:** Median Percentage Change From BL in T-Cell Subpopulation Ratios: SAD vs Non-SAD Cohorts

Entire cohort (n = 130)			
	SAD (n = 38)	No-SAD (n = 92)	
Variable	Median (IQR)	Median (IQR)	*p* Value^a^
CD4^+^ Trte:CD4			
BL (raw value)	0.26 (0.12 to 0.34)	0.31 (0.23 to 0.42)	0.04
Percentage change M6 comp. BL	−12% (−39 to 27)	−14% (−38 to 5)	0.59
Percentage change M12 comp. BL	19% (−12 to 80)	−3% (−24 to 27)	0.04
Percentage change M24 comp. BL	48% (2 to 82)	−5% (−28 to 24)	0.001
CD4^+^ Trte:CD8+ Temra			
BL (raw value)	1.73 (0.76 to 4.39)	3.61 (1.47 to 7.24)	0.02
Percentage change M6 comp. BL	−60% (−76 to −23)	−54% (−80 to −21)	0.94
Percentage change M12 comp. BL	−5% (−42 to 119)	−32% (−67 to 20)	0.03
Percentage change M24 comp. BL	100% (−29 to 236)	−23% (−77 to 25)	<0.001
CD4^+^ Treg:CD8+ Temra			
BL (raw value)	0.72 (0.22 to 1.54)	0.96 (0.42 to 1.54)	0.29
Percentage change M6 comp. BL	−16% (−67 to 42)	−26% (−63 to 13)	0.54
Percentage change M12 comp. BL	2% (−42 to 85)	−25% (−43 to 87)	0.10
Percentage change M24 comp. BL	20% (−15 to 116)	−16% (−53 to 17)	<0.01
CD3:CD19			
BL (raw value)	3.95 (2.67 to 7.00)	4.46 (3.10 to 6.62)	0.59
Percentage change M6 comp. BL	−68% (−82 to −46)	−10% (−67 to 68)	<0.001
Percentage change M12 comp. BL	−51% (−74 to −39)	−33% (−52 to 36)	0.03
Percentage change M24 comp. BL	−57% (−71 to −19)	−34% (−58 to 16)	0.03

ALZ (n = 51)			
Variable	SAD (n = 23)	Non−SAD (n = 28)	
CD4^+^ Trte:CD8+Temra	Median (IQR)	Median (IQR)	*p* Value
BL (raw value)	1.34 (0.72 to 2.93)	3.45 (1.55 to 5.97)	0.02
Percentage change M6 comp. BL	−60% (−77 to −13)	−66% (−87 to −31)	0.42
Percentage change M12 comp. BL	11% (−44 to 127)	−32% (−74 to 72)	0.15
Percentage change M24 comp. BL	123% (41 to 327)	−21% (−68 to 50)	0.03

CladT (n = 59)			
Variable	SAD (n = 6)	Non−SAD (n = 53)	
CD4^+^ Trte:CD8+ Temra	Median (IQR)	Median (IQR)	*p* Value
BL (raw value)	4.39 (1.08 to 7.60)	5.16 (1.84 to 11.4)	0.45
Percentage change M6 comp. BL	−55% (−75 to −43)	−38% (−69 to −8)	0.37
Percentage change M12 comp. BL	−27% (−42 to −8)	−43% (−66 to −12)	0.42
Percentage change M24 comp. BL	−62% (−84 to −24)	−38% (−77 to −2)	0.39

Abbreviations: ALZ = alemtuzumab; BL = baseline; CladT = cladribine tablets; comp. = compared with; IQR = interquartile range; M = month; SAD = secondary autoimmune disease; Temra = terminally differentiated effector memory; Treg = T regulatory cell; Trte = T-cell recent thymic emigrant.

a Mann-Whitney *U* test. A full version of this table is given in eTable 4.

#### Impact of Lymphocyte Subpopulations on the Risk of Disease Activity

In a multivariable analysis, the BL CD8^+^ T-cell count (aHR 0.34, 95% CI 0.15–0.80, *p* = 0.01) was associated with a reduced hazard of EDA-3 (Table 4 and eTable 5). No other significant associations were observed among the investigated lymphocyte subpopulations and T-cell subpopulation ratios.

**Table 4:** Cox Regression Models for BL Lymphocyte Subpopulations and the Prediction of Disease Activity (EDA-3)

	Univariable model			Cox proportional hazards		
Variable	HR	95% CI	*p* Value	aHR	95% CI	*p* Value
Lymphocytes tot.	0.83	0.63–1.10	0.19	0.76	0.56–1.03	0.07
CD3^+^ T cells	0.82	0.57–1.19	0.30	0.74	0.50–1.11	0.14
CD4^+^ T cells	1.01	0.53–1.93	0.98	0.86	0.42–1.80	0.72
CD8^+^ T cells	0.44	0.21–0.96	**0.04**	0.34	0.15–0.80	**0.01**
NK cells	0.41	0.08–2.15	0.30	0.40	0.08–1.97	0.26
CD19^+^ B cells	0.75	0.27–2.11	0.58	0.56	0.19–1.66	0.29
CD3:CD19	1.04	0.96–1.13	0.38	1.06	0.97–1.16	0.19
TRECs	1.00	1.00–1.00	0.71	1.00	1.00–1.00	0.46
CD4^+^ Treg:CD4	71	0.01–6 × 10^5^	0.36	34	0.01–2 × 10^6^	0.51
CD4^+^ Trte:CD4	2.37	0.41–13.7	0.34	1.73	0.21–14.6	0.61
CD8^+^ Temra:CD8	0.34	0.06–1.96	0.23	0.35	0.06–2.24	0.27
CD4^+^ Trte:CD8^+^ Temra	1.02	0.98–1.04	0.09	1.02	0.99–1.04	0.13
CD4^+^ Treg:CD8^+^ Temra	1.16	1.00–1.34	0.05	1.15	0.99–1.33	0.07

Abbreviations: aHR = adjusted hazard ratio; BL = baseline; CI = confidence interval; DMT = disease-modifying therapy; EDA-3 = evidence of disease activity; HR = hazard ratio; NK = natural killer; Temra = terminally differentiated effector memory; tot. = total; TRECs = T-cell receptor excision circles; Treg = T regulatory cell; Trte = T-cell recent thymic emigrant.

Cox proportional hazard regression analysis showing the association between immune cell subpopulations and EDA-3. Univariable models present HRs, 95% CIs, and *p* values for each variable analyzed individually. Multivariable models (aHR) account for potential confounders (age, sex, disease duration, MRI T2 lesion burden, and number of previous DMTs). An HR or aHR <1 indicates a protective effect while HR or aHR >1 suggests increased risk. Statistically significant associations are highlighted in bold.

Details on confounder variables used in the multivariable models are provided in eTable 5.

## Discussion

In this observational cohort study, we compared lymphocyte dynamics using a comprehensive flow cytometry panel after treatment with ALZ, CladT, and AHSCT during the first 2 years following the initiation of IRT. We assessed, across cohorts, their association with disease recurrence and SAD over a median follow-up of 4.7 years. This study applies a multiparameter immunophenotyping panel consecutively across cohorts of 3 major IRTs, whereas most previous studies have examined individual treatments in the context of randomized controlled trials (RCTs).^11,12^ We confirm that lymphocyte depletion and repopulation differed significantly across IRT cohorts. While AHSCT-treated patients recovered their lymphocyte subpopulations most rapidly, depletion of T-cell subsets was most pronounced and sustained after ALZ treatment and CladT-treated patients had the slowest repopulation of CD19^+^ B cells. A low CD4^+^ Trte:CD8+ Temra ratio at BL (hypothetically indicating an activated state), followed by a sharp increase at months 12 and 24, appears to be associated with the development of SAD. No definitive associations were found between lymphocyte subpopulations and disease activity, although a lower BL CD8^+^ T-cell value may suggest an increased risk.

The observed differences in lymphocyte depletion and repopulation among the 3 IRT cohorts are expected, given the distinct mechanisms of action underlying each treatment. While ALZ targets CD52, expressed on circulating T and B cells,^13^ CladT disrupt DNA synthesis and repair, causing strand breaks, apoptosis, and preferential T-cell and B-cell depletion.^14,15^ AHSCT provides the most comprehensive immune reset and is administered as a single treatment,^16^ in contrast to the intermittent dosing of ALZ and CladT over 2 years. Of interest, CladT induced a longer duration of lymphocyte depletion than AHSCT, despite generally being considered a moderate-efficacy DMT compared with the high efficacy of AHSCT.^17,18^ A similar pattern was seen with ALZ, yet AHSCT remains superior in clinical outcomes,^19^ suggesting that depletion duration alone may not determine efficacy. The depth of initial lymphocyte depletion may be more relevant. It is reasonable to assume that the AHSCT cohort would have exhibited the lowest lymphocyte subpopulation levels compared with the ALZ and CladT cohorts if blood sampling had been conducted earlier, within 3 months of initiating IRT. Alternatively, the broader impact of AHSCT on the innate immune system,^17^ an aspect that is less affected by CladT^14,15^ and ALZ^13^ treatment, may contribute to its superior effectiveness. Furthermore, the integration of intensive immune ablation (by cyclophosphamide) with accelerated reconstitution through stem cell infusion differentiates AHSCT from other 2 IRTs and may contribute to its therapeutic efficacy.

Among our 3 IRT cohorts, 57% (ALZ), 15% (AHSCT), and 10% (CladT) developed a SAD. This is slightly higher than previously reported for ALZ (48%),^3^ but on par with the range reported for AHSCT (4%–17%).^20^ CladT have not been associated with an increased risk of SAD.^2^ Nevertheless, following a case of anti-glomerular basement membrane antibody–mediated glomerulonephritis after the second treatment in year 2, the possibility of SAD in CladT-treated patients cannot be completely excluded.^21^ However, a United Kingdom–based study found that autoimmune disorders affect approximately 10% of the general population,^22^ a prevalence similar to that observed in our CladT cohort.

We used T-cell subpopulation ratios to assess changes in the proportions of various T-cell subpopulations relative to total CD4^+^, CD8^+^, and CD8^+^ Temra populations and to explore their potential association with SAD. We show that the SAD cohort had lower median BL CD4^+^ Trte:CD4 and CD4^+^ Trte:CD8+ Temra ratios compared with the non-SAD cohort. The CD4^+^ Trte:CD4 ratio reflects thymic-derived immature T cells while the CD4^+^ Trte:CD8+ Temra ratio represents thymic output relative to effector cells (i.e., an indicator of the balance between T helper [CD4^+^] and cytotoxic [CD8^+^] T-cell compartments in the immune system). Therefore, a higher value for each ratio could be hypothesized to reduce SAD risk, which aligns with our BL results. By contrast, during follow-up, the SAD cohort showed a more reactive pattern with a greater relative increase in these 2 ratios, as well as in the CD4^+^ Treg:CD8+ Temra ratio, compared with the non-SAD group. The CD4^+^ Treg:CD8+ Temra ratio reflects the proportion of regulatory T cells relative to effector cells. Contrary to the hypothesis that these ratios would be protective, we observed a greater increase in these 3 ratios during follow-up in the SAD group compared with the non-SAD group. The difference was most pronounced for the CD4^+^ Trte:CD8+ ratio, which was confirmed in the ALZ cohort, but not in the CladT cohort. This suggests that CD4^+^ Trte, rather than being protective, may be more autoreactive. In this case, SAD could arise in the thymus, indicating a failure of central tolerance and suggesting that ALZ affects the thymus differently than the other treatments, particularly CladT. It has been reported that ALZ directly targets thymic epithelial cells and inhibits thymopoiesis, resulting in a prolonged dependence on peripheral expansion for immune reconstitution.^13^ By contrast, CladT do not directly target thymic epithelial cells or thymocytes, resulting in a less severe, indirect effect on thymic function.^23-26^

No significant differences in the median CD19^+^ B-cell count and CD3:CD19 ratio were found at BL among the 3 IRT cohorts. However, we show that CladT-treated pwMS had lower median CD19^+^ B-cell counts and CD3:CD19 ratios at months 6, 12, and 24 compared with ALZ-treated pwMS. Among IRTs, ALZ is associated with the highest incidence of SADs, primarily autoimmune autoantibody-mediated (i.e., B cell–dependent) AITDs.^3^ By contrast, CladT have not been associated with an increased risk of SADs.^1,2^ This supports existing data that the higher incidence of SADs in patients with MS treated with ALZ, compared with CladT, is primarily due to differences in immune reconstitution mechanisms and immune cell recovery dynamics. While ALZ causes significant depletion of lymphocytes, particularly CD4^+^ and CD8^+^ T cells, with prolonged suppression and rapid hyper-repopulation of naive B cells,^11,13,27,28^ CladT induce a more modest depletion of T cells and prolonged depletion of B cells.^23,24,29^ These milder, more balanced immune effects, coupled with the absence of B-cell hyper-repopulation, are believed to reduce the risk of SADs in CladT-treated pwMS.

We found no definitive association between lymphocyte subpopulations and disease activity; however, our results suggest that a lower BL CD8^+^ T-cell count may be indicative of an increased risk. Contrary to our findings, CD8^+^ T cells have been shown to be pathogenic in experimental autoimmune encephalomyelitis (EAE; i.e., the most commonly used experimental model for MS), either independently or by exacerbating CD4^+^ T cell–driven disease, despite EAE's traditional classification as a CD4^+^ T cell–mediated model.^30-32^ However, emerging data suggest a regulatory role for CD8^+^ T cells in MS pathogenesis.^33^ In addition, CD8^+^ T-cell depletion prior to EAE induction exacerbates the disease.^34^ Likely, CD8^+^ T-cell subsets exhibit different effector functions in the context of MS/EAE.

This study has some limitations. First, included patients have been previously either untreated or treated with DMTs with different modes of action. In addition, combining data from multiple cohorts (e.g., ALZ, AHSCT, and CladT) may introduce heterogeneity because of differences in cohort characteristics. To address this, we excluded patients who either (1) had total lymphocyte counts below the WHO grade 2 lymphopenia threshold (<0.8 x 10e9/L) or (2) received B cell–depleting therapy as their last treatment before starting IRT. In addition, we used harmonized data collection protocols to minimize inconsistencies. Second, CD19^+^ B cells were analyzed as part of the TBNK panel, and a comprehensive B-cell panel was not performed; therefore, data on other B-cell subtypes were unavailable. Third, owing to the small size of the AHSCT cohort, the results for this group should be interpreted with caution, given the increased risk of type II error. Fourth, we analyzed repeated measures using cross-sectional comparisons at each time point, without modeling intraindividual changes over time. This may have limited our ability to detect longitudinal trends. Future studies could benefit from applying repeated-measures models to better capture immune dynamics. Finally, follow-up time varied across the 3 IRT cohorts. However, most reconstitution occurs within the first 2 years after the start of IRT. In addition, the occurrence of SAD peaks during years 2–3 after IRT. All cohorts were followed beyond these time frames (median follow-up: 4.7 years). Furthermore, statistical corrections for follow-up time were applied in the EDA-3 analyses, ensuring that differences in follow-up time did not significantly influence our results.

In conclusion, lymphocyte depletion and repopulation differ substantially across IRTs. AHSCT leads to the fastest recovery, ALZ induces the most durable T-cell depletion, and CladT result in the slowest CD19^+^ B-cell repopulation. A low BL CD4^+^ Trte:CD8+ Temra ratio, followed by a marked increase over time, may be associated with the development of SAD. Although no clear associations were found between lymphocyte subpopulations and disease activity, a lower BL CD8^+^ T-cell count may suggest increased risk and warrants further investigation.

## Glossary

aHR: adjusted hazard ratio
AHSCT: autologous hematopoietic stem cell transplantation
AITD: autoimmune thyroid disease
ALZ: alemtuzumab
BL: baseline
CDW: confirmed disability worsening
CI: confidence interval
CladT: cladribine tablets
DMT: disease-modifying therapy
DNA: deoxyribonucleic acid
EAE: experimental autoimmune encephalomyelitis
EDA-3: evidence of disease activity
EDSS: Expanded Disability Status Scale
IRT: immune reconstitution therapy
IQR: interquartile range
MS: multiple sclerosis
NEDA-3: no evidence of disease activity
NK: natural killer
pwMS: people with multiple sclerosis
RRMS: relapsing-remitting multiple sclerosis
SADs: secondary autoimmune diseases
SD: standard deviation
Tcm: T-cell central memory
Tem: T-cell effector memory
Temra: terminally differentiated effector memory
Tnaive: T-cell naive
TRECs: T-cell receptor excision circles
Treg: T regulatory cell
Trte: T-cell recent thymic emigrant
VLA-4: very late antigen 4
WHO: World Health Organization
